# Successful temporary resection of a ruptured hepatoblastoma without preoperative chemotherapy: A case report presenting a novel surgical strategy

**DOI:** 10.1016/j.ijscr.2020.09.202

**Published:** 2020-10-10

**Authors:** Masaki Horiike, Maya Sogabe, Sinchul Jwa, Sadao Tokimasa, Shoji Kubo

**Affiliations:** a Department of Pediatric Surgery, Japanese Red Cross Society Wakayama Medical Center, 4-20, Komatsubara-dori, Wakayama City, Japan; b Department of Pediatrics, Osaka City University Graduate School of Medicine, 1-4-3, Asahimachi, Abenoku, Osaka, 545-8585, Japan; c Department of Hepato-Biliary-Pancreatic Surgery, Osaka City University Graduate School of Medicine, 1-4-3, Asahimachi, Abenoku, Osaka, 545-8585, Japan

**Keywords:** Hepatoblastoma, Lung metastases, Tumor rupture, Emergency surgery, Temporary tumor resection, Case report

## Abstract

**Introduction:**

Although spontaneous tumor rupture is a serious complication of hepatoblastoma, there is no consensus regarding the treatment strategy in infants. We report a patient with lung metastases who had a ruptured hepatoblastoma prior to the start of the scheduled chemotherapy and was successfully treated with a combined treatment including liver resection, lung resection, and chemotherapy.

**Presentation of case:**

A 22-month-old boy with a ruptured hepatoblastoma and lung metastases underwent an emergency laparotomy with complete tumor resection, followed by chemotherapy. Moreover, a barely detectable metastatic lung lesion shown by a chest CT scan was resected after the fifth chemotherapy treatment. Both postoperative and chemotherapy courses were uneventful. The patient survived without any recurrent hepatoblastoma 2 years after the emergency surgery despite the poor prognosis indicated by distant metastases at the time of diagnosis.

**Discussion:**

Because rupture itself can be the main cause of death in patients with hepatoblastoma, emergency tumor hemostasis is essential. However, there are no reports comparing the prognosis of the treatment method performing tumor hemostasis alone, tumor resection after chemotherapy, and tumor hemostasis and resection at the same time. The clinical course of the patient indicates that performing tumor hemostasis and resection simultaneously and lung resection after chemotherapy is an effective option to treat a ruptured hepatoblastoma with disseminated tumors and lung metastases if the patient’s condition is stable.

**Conclusion:**

Aggressive treatment with surgery and chemotherapy is an effective option for ruptured hepatoblastoma with disseminated tumors and lung metastases in infants.

## Highlights

• There is no consensus regarding the treatment strategy of ruptured hepatoblastoma in infants.
• The patient was shocked by the rupture of tumor, and hemostasis and tumor resection were performed at the same time by emergency laparotomy.
• There are no reports comparing the prognosis of tumor hemostasis alone and tumor hemostasis and resection at the same time.
• The patient remained in remission 2 years after emergency surgery despite the poor prognosis case.
• Aggessive treatment with tumor resection and lung resection and chemotherapy is an effective option for ruptured hepatoblastoma.

## 1 Introduction

Hepatoblastoma is a rare malignant tumor that accounts for only approximately 1% of all pediatric cancers and is most effectively treated with complete resection. However, it is often diagnosed at advanced stages when resection is impossible or distant metastasis has already occurred. Recently, multidisciplinary protocols with both surgery and chemotherapy have been established for the treatment of hepatoblastoma [1].

Spontaneous rupture of the tumor is a serious complication of hepatoblastoma, and various treatments such as liver resection, transcatheter arterial embolization (TAE), and chemotherapy have been attempted [2,3]. However, there is no consensus regarding the treatment strategy in infants. Recently, we had a patient with lung metastases who had a ruptured hepatoblastoma prior to the start of the scheduled chemotherapy and was successfully treated with a combined treatment including liver resection, lung resection, and chemotherapy. In this report, we propose an aggressive treatment with surgery and chemotherapy as an effective option for ruptured hepatoblastoma with disseminated tumors and lung metastases. This work was reported in line with the SCARE 2018 criteria [4]. The Research Registry UIN is 5920.

## 2 Presentation of case

**Fig. 1:**
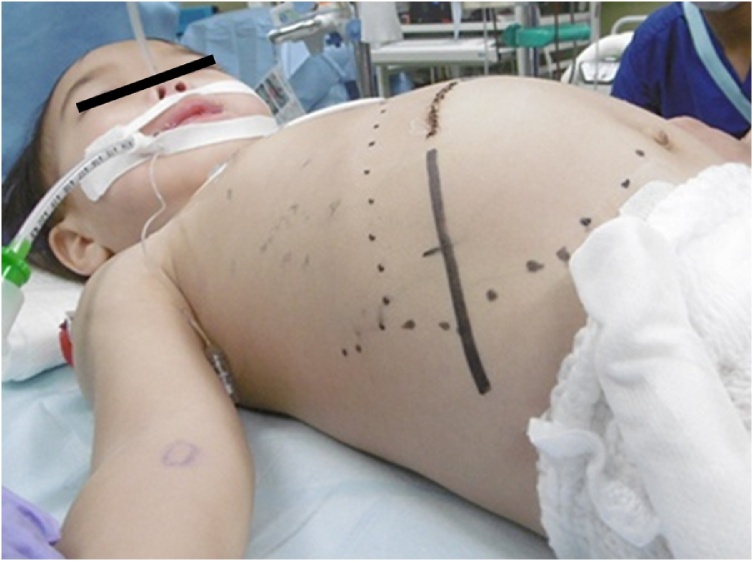
Abdominal macroscopic findings of the patient.

**Fig. 2:**
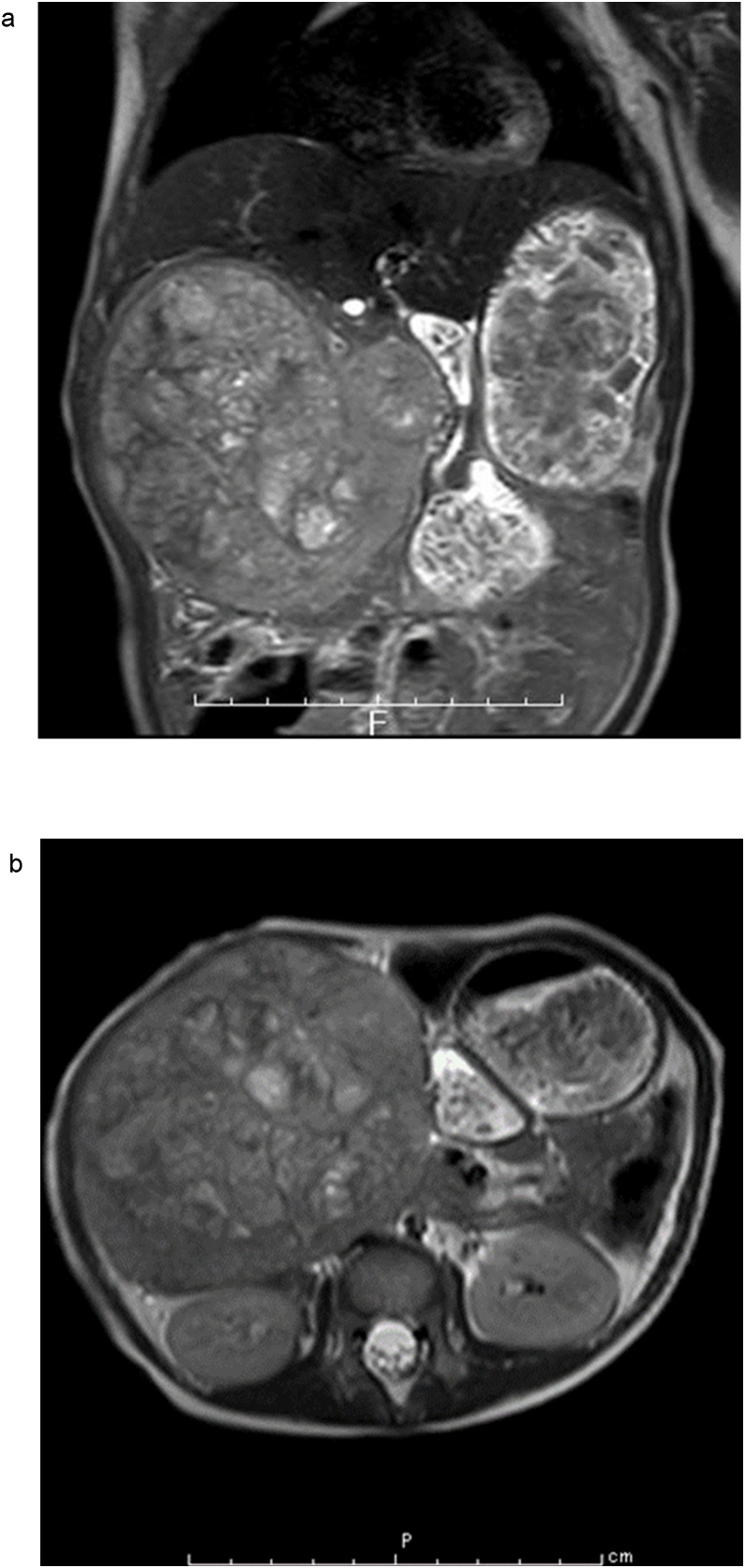
a,b: Abdominal magnetic resonance imaging (T2weighted) showing a large solid tumor approximately 100 mm in diameter protruding into the abdominal cavity from the right hepatic lobe.

**Fig. 3:**
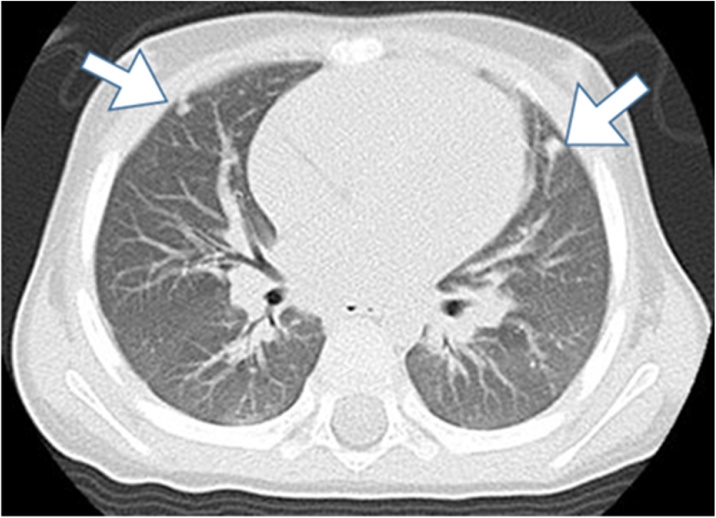
Chest computed tomography showing parenchymal nodules suspicious for metastases in both lungs.

A 22-month-old boy with the chief complaint of an abdominal mass was referred to our hospital. An elastic, hard mass was palpable in his right abdomen on physical examination (Fig. 1). Ultrasonography revealed a nonuniform, hyperechoic mass in the right lobe of the liver. Abdominal magnetic resonance imaging revealed a large solid tumor, with a heterogeneous interior on T2-weighted images, approximately 100 mm in diameter, protruding into the abdominal cavity from the right hepatic lobe (Fig. 2). Chest computed tomography (CT) revealed parenchymal nodules considered to be metastases in both lobes of the lungs (Fig. 3). Although liver function test results were within normal ranges, serum α-fetoprotein (AFP) concentration was 248,443 ng/mL. A mixed embryonal/fetal hepatoblastoma was diagnosed via liver tumor biopsy.

**Fig. 4:**
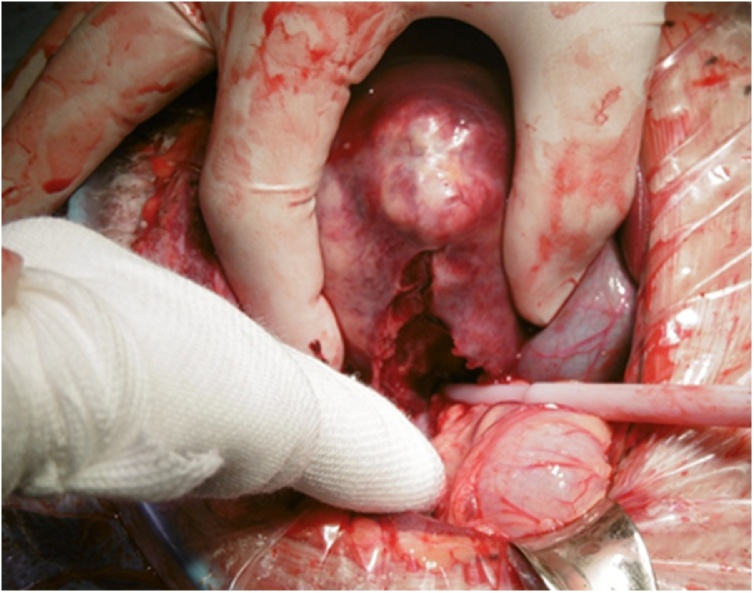
The rupture site on the left side of the tumor.

**Fig. 5:**
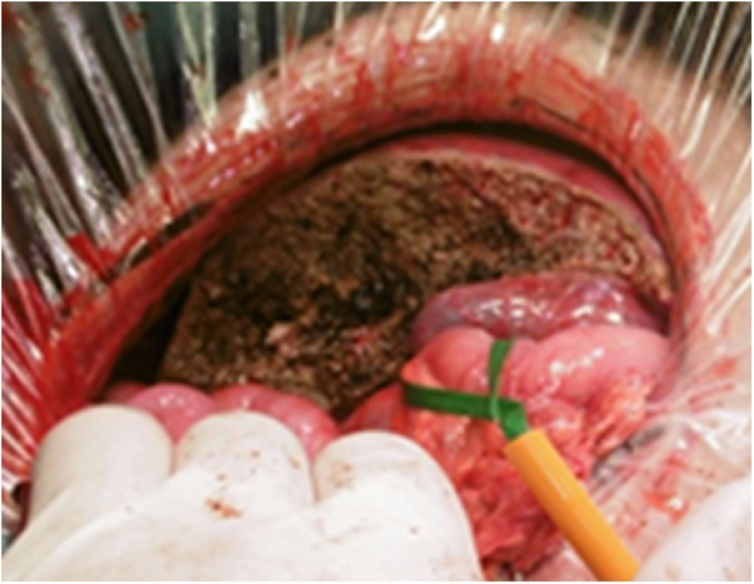
Findings of complete removal of tumor protruding from segments 5,6.

We planned chemotherapy to treat the hepatoblastoma and lung metastases. However, the day before the scheduled start of chemotherapy, the patient became lethargic and tachycardic, with unstable vital signs that led to shock that was unresponsive to conservative measures. His hemoglobin level was 6.8 g/L. We performed an emergency laparotomy because the patient’s small size precluded TAE. During the surgery, the left margin of the tumor ruptured and the tumor contents leaked into the abdominal cavity (Fig. 4). The patient’s respiratory and circulatory statuses stabilized after blood transfusion and hemostatic compression of the tumor rupture site. The tumor protruded from the liver, and the capsule had also thinned in other areas that appeared to be in a state of imminent rupture. Therefore, we performed a partial resection of the liver (segments 5 and 6) along the boundary between the tumor and the healthy liver (Fig. 5). In addition, all visible tumor contents in the abdominal cavity were removed. The total operation time was 2 h 54 min, and the intraoperative blood loss volume was 1,190 mL. The postoperative course was uneventful. The histological results for the resected tumor were the same as those from the biopsies.

**Fig. 6:**
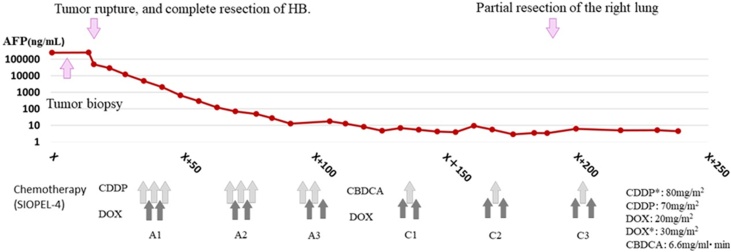
Chemotherapy treatment course (modified SIOPEL-4 regimen) and AFP trends.

The patient received six courses of chemotherapy (modified SIOPEL-4 regimen) in the pediatric department (Fig. 6). Chest CT performed after chemotherapy showed a barely detectable metastatic lesion in the right lung, which was resected after the fifth chemotherapy treatment, as a reference to the protocol of the SIOPEL-4 regimen. Serum AFP level had decreased logarithmically and remained <10 ng/mL after the completion of chemotherapy. The patient remained in remission 2 years after the emergency surgery despite the poor prognosis indicated by distant metastases at the time of initial diagnosis.

## 3 Discussion

In recent years, surgical resection with pre- and/or post-operative chemotherapy has markedly improved the survival rate of patients with hepatoblastoma [5]. However, hepatoblastoma is sometimes detected at an advanced stage. Rupture of hepatoblastoma occurs in 2.9–8.6% of patients [3], and it is the main cause of death in patients with hepatoblastoma (40%) [6]. In the present patient, an emergency laparotomy was necessary because the tumor ruptured prior to the start of scheduled chemotherapy for hepatoblastoma and lung metastases. Several reports have indicated the usefulness of liver resection with chemotherapy for ruptured hepatoblastoma [2,3,6]. Therefore, we resected the liver and removed the disseminated tumors, which was followed by a course of chemotherapy. In addition, the remnant lung metastasis after chemotherapy was also resected. The resected lung metastatic lesion was viable by pathological search, but other lung metastatic lesions were not visible on the image, suggesting that this chemotherapy was effective. As a result, the patient survived without any evident hepatoblastoma. There are no reports comparing the prognosis of the treatment method in which tumor hemostasis is preceded while the tumor is resected after chemotherapy and the treatment method in which tumor hemostasis and resection are performed at the same time. The clinical course of this patient indicates that an aggressive treatment method, in which tumor hemostasis and resection are performed at the same time and lung resection is performed after chemotherapy, is an effective option to treat a ruptured hepatoblastoma with disseminated tumors and lung metastases if the patient’s condition is stable.

## 4 Conclusion

We are focusing on the usefulness of temporary tumor resection at the time of rupture as well as the removal of tumor contents that have disseminated intraperitoneally while still being confined. This type of procedure can lead to a good clinical course in young patients. Even if the vital signs deteriorate temporarily at the time of tumor rupture, it may be possible to resect the tumor temporarily if vital signs stabilize after the start of conservative treatment.

Although there is no consensus on the treatment of ruptured hepatoblastoma in infants, we believe it is important to actively treat hepatoblastoma cases that include distant metastases.

## Declaration of Competing Interest

All authors declare that they have no conflicts of interest.

## Funding

All authors declare that they have no funding source.

## Ethical approval

This submission is a case report about surgical strategy, not the manuscript reporting studies involving human participants, human data or human tissue.

In this case, ethical approval has been exempted by our institution.

## Consent

Written informed consent was obtained from the patient for publication of this case report and accompanying images. A copy of the written consent is available for review by the Editor-in-Chief of this journal on request.

## Author contribution

Masaki Horiike (MH) made the conception and design of this case report. Authors other than MH contributed to the collection, analysis, and interpretation of the data. MH wrote the draft manuscript, and other authors performed the critical revision of the manuscript. All authors gave final approval of the version to be published. MH has overall responsibility and guarantees the scientific integrity.

## Registration of research studies

1. Name of the registry: Masaki Horiike.
2. Unique identifying number or registration ID: Researchregistry5920.
3. Hyperlink to your specific registration (must be publicly accessible and will be checked): https://www.wakayama-med.jrc.or.jp/department/shonigeka/.

## Guarantor

MH has overall responsibility and guarantees the scientific integrity.

## Provenance and peer review

Not commissioned, externally peer-reviewed.
